# Metallization without Charge Transfer in CuReO_4_ Perrhenate under Pressure

**DOI:** 10.1021/acs.inorgchem.4c05051

**Published:** 2025-03-20

**Authors:** Daria Mikhailova, Stanislav M. Avdoshenko, Maxim Avdeev, Michael Hanfland, Ulrich Schwarz, Yurii Prots, Angelina Sarapulova, Konstantin Glazyrin, Leonid Dubrovinsky, Anatoliy Senyshyn, Jens Engel, Helmut Ehrenberg, Alexander A. Tsirlin

**Affiliations:** † Leibniz Institute for Solid State and Materials Research Dresden (IFW Dresden), Helmholtzstr. 20, D-01069 Dresden, Germany; ‡ Australian Nuclear Science and Technology Organisation, Lucas Heights, NSW 2234, Australia; § School of Chemistry, The University of Sydney, Sydney, NSW 2006, Australia; ∥ European Synchrotron Radiation Facility, 71 Av. des Martyrs, 38000 Grenoble, France; ⊥ Max Planck Institute for Chemical Physics of Solids, Nöthnitzer Str. 40, D-01187 Dresden, Germany; # Freiburg Materials Research Center (FMF), Stefan-Meier-Straße 21, 79104 Freiburg, Germany; ∇ Fraunhofer Institute for Solar Energy Systems, Heidenhofstr. 2, 79110 Freiburg, Germany; ○ Bavarian Research Institute of Experimental Geochemistry and Geophysics, University of Bayreuth, Universitätsstr. 30, D-95440 Bayreuth, Germany; ◆ Deutsches Elektronen-Synchrotron (DESY), Notkestr. 85, 22607 Hamburg, Germany; ¶ Forschungsneutronenquelle Heinz Maier-Leibnitz FRM-II, Technische Universität München, Lichtenbergstr. 1, D-85747 Garching near München, Germany; ⋈ Institut für Werkstoffwissenschaft, Technische Universität Dresden, Helmholtzstr. 7, D-01062 Dresden, Germany; ⧓ Karlsruhe Institute of Technology (KIT), Institute for Applied Materials (IAM), Hermann-von-Helmholtz-Platz 1, D-76344 Eggenstein-Leopoldshafen, Germany; ⧖ Felix Bloch Institute for Solid-State Physics, Leipzig University, 04103 Leipzig, Germany; ● Experimental Physics VI, Center for Electronic Correlations and Magnetism, University of Augsburg, 86135 Augsburg, Germany

## Abstract

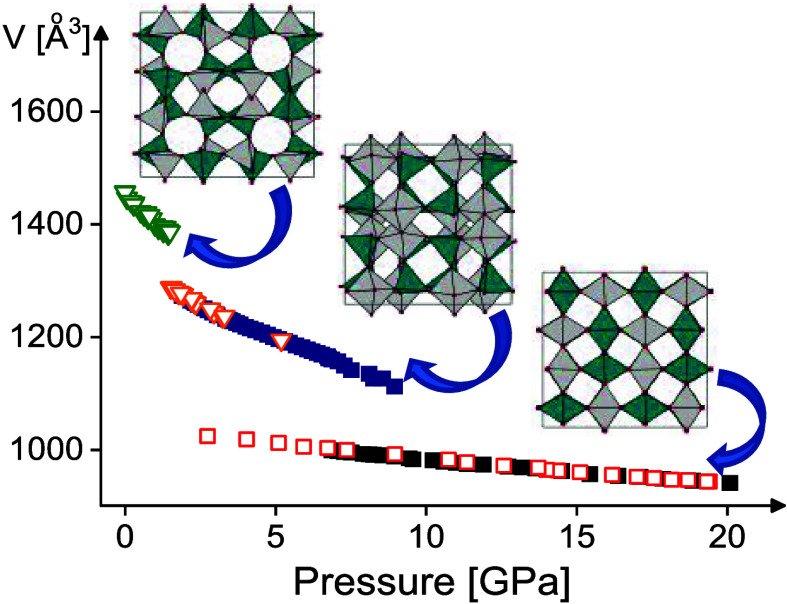

Using high-pressure synchrotron X-ray diffraction combined with Raman spectroscopy and density-functional calculations, we determined the sequence of the pressure-induced transformations in CuReO_4_. At 1.5 GPa, the lattice symmetry changes from *I*4_1_*cd* to *I*4_1_/*a* with the transformation of isolated ReO_4_-tetrahedra into infinite chains of ReO_6_-octahedra. The second, isosymmetric transition at 7 GPa leads to the formation of a NbO_2_-type structure with the octahedral oxygen coordination for both Cu^1+^ and Re^7+^ cations. Both transitions are of the first order and accompanied by discontinuities in the unit-cell volume of 7 and 14%, respectively. Density-functional calculations predict the metallic state of the high-pressure NbO_2_-type phase of CuReO_4_, and this prediction is in-line with the disappearance of the Raman signal above 7 GPa and visual observations (darkness/reflection of the sample). This metallization is caused by the increased bandwidth of both Cu 3d and Re 5d bands without any significant charge transfer between Cu and Re. At ambient pressure, the crystal structure of CuReO_4_ is retained between 4 and 700 K (melting point), showing a negative thermal expansion along the *c*-axis and a positive expansion along the *a*-axis within the entire temperature range.

## Abstract 2

Using high-pressure synchrotron X-ray diffraction combined with Raman spectroscopy and density-functional calculations, we determined the sequence of the pressure-induced transformations in CuReO_4_.

## 1 Introduction

Pressure-induced insulator-to-metal transitions in oxides represent a fascinating topic in solid-state science. Such transitions are often accompanied by charge transfer. Valence changes under pressure facilitate the formation of unconventional oxidation states and peculiar electronic structures.^1,2^ The pressure-induced reduction of Re^7+^ in complex oxides containing a 3d metal would enable experimental access to lower oxidation states of Re with a partially filled 5d shell and an intricate manifestation of correlation effects combined with strong spin–orbit coupling. Additionally, the pressure-induced reduction of Re^7+^ implies a strong electronic coupling between the Re atoms and the oxidized 3*d* element, thus leading to an additional flexibility of the electronic system.

Many ternary oxides with Re in the oxidation states between +4 and +6 and a 3d transition metal can be synthesized under high-pressure high-temperature conditions, for example, rutile-like FeRe_2_O_6_, CoRe_2_O_6_, and NiRe_2_O_6_ with mixed Re^4+^ and Re^6+^ states,^3^ rutile-like CoReO_4_ with the ordered arrangement of the Co and Re cations,^4^ wolframite-like MnReO_4_ with Mn^2+^ and Re^6+^,^5^ double perovskite Mn_3_ReO_6_,^6^ or rutile-like V_0.5_Re_0.5_O_2_, which presumably contains V^4+^ and Re^4+^.^7^

The lower oxidation states of Re in oxides with 3d transition metals may also be adopted at elevated temperatures under normal pressure or even in vacuum, for example, in Sc_6_ReO_12_ with a distorted fluorite-type structure^8^ or in rutile-type compound ScRe_2_O_6_^9^ and Cr_*x*_Re_1–*x*_O_2_.^10^ Rutile-type solid-solutions V_1–*x*_Re_*x*_O_2_ with 0.01 ≤ *x* ≤ 0.30 can also be prepared via a solid-state synthesis in evacuated sealed silica tubes.^11^ Depending on the composition, Re^4+^, Re^6+^, or a combination thereof have been found in the material.^11^ But generally, ternary compounds that combine 3d transition metals with Re in an intermediate valence state are rarely stabilized at ambient pressure.

Several perrhenates were extensively studied under high pressure in the search for the insulator-to-metal transition and concomitant charge transfer. Ag^+^Re^7+^O_4_ and Tl^+^Re^7+^O_4_ indeed show a sequence of high-pressure transformations,^12−16^ but the evolution of the Re valence across these transitions remains controversial.^13,16,17^ No conclusive evidence of metallization could be obtained either. At ambient pressure, TlReO_4_ also demonstrates temperature-induced structural phase transitions without any valence change.^18,19^ From a chemical point of view, the redox potential of the Re^7+^/Re^5+^ pair may be too low to oxidize Ag^+^ or Tl^+^ toward the valence state of +3. A more promising candidate for pressure-induced oxidation is Cu^+^, which is likely to transform into the more stable Cu^2+^-state, especially under oxidative conditions. Cu^+^Re^7+^O_4_ has been characterized at ambient pressure,^20^ but its evolution under pressure has not been studied yet.

In contrast to other perrhenates with the scheelite structure, CuReO_4_ (space group (SG) *I*4_1_*cd*) adopts its own structure type^20^ with a three-dimensional framework of corner-sharing CuO_4_ and ReO_4_ tetrahedra, similar to some silicon oxide and aluminosilicate structures. The structure conforms to the Loewenstein-rule.^21^ Each ReO_4_ and CuO_4_ tetrahedron is surrounded by 4 CuO_4_ and ReO_4_ tetrahedra, respectively, while the tetrahedra of the same element is never directly connected to each other. Along the *c*-axis, the structure exhibits 4-fold double chains known from the mineral Narsarsukite.^22^ There are also 6-, 8-, and 10-fold spirally twisted rings of the coordination polyhedra in the structure.^20^ Large channels with an average diameter of 3.1 Å along the *c*-axis, large enough for the penetration of gaseous O_2_ and H_2_O molecules, may facilitate the rapid decomposition of CuReO_4_ in air. These channels might also promote pressure- and temperature-induced structural changes. According to numerous experimental studies, high-pressure polymorphs of complex silicon oxides crystallize in different structure types.^23^

In the present work, we study the evolution of the CuReO_4_ structure as a function of temperature at ambient pressure and as a function of pressure at room temperature in the search for structural phase transitions and possible valence change. No transitions were observed as a function of temperature, although a highly anisotropic thermal expansion was revealed. By contrast, two abrupt transformations are observed under moderate pressures of about 1.5 and 6.7 GPa. These phase transitions are characterized by the increase in the coordination number from 4 to 6 for Re and Cu, respectively. The second transition corresponds to the metallization of CuReO_4_.

## 2 Experimental Section

### 2.1 Synthesis and Thermal Stability Studies of the Ambient-Pressure Polymorph CuReO_4_–AP

CuReO_4_ was synthesized in a sealed silica tube at 773 K using Cu_2_O and Re_2_O_7_ (both oxides from Alfa Aesar, 99.9%), according to the method described in ref (20). Phase purity was evaluated using powder X-ray diffraction (XRD) with a STOE STADI P diffractometer (Cu Kα_1_-radiation, λ = 1.54059 Å) in steps of 0.02° for 2θ from 3 to 90° in the transmission mode.

The temperature evolution of the CuReO_4_ crystal structure has been investigated by synchrotron X-ray powder diffraction at HASYLAB/DESY (Hamburg, Germany) at the beamline B2.^24^ The measurements were performed in the Debye–Scherrer mode using the on-site readable image-plate detector OBI^25^ and a He closed-cycle cryostat^26^ or a STOE furnace equipped with a EUROTHERM temperature controller and a capillary spinner. The data were collected upon heating in steps of 0.004° over the 5–45° 2θ range with a temperature increment of 20 K between 100 and 700 K with the wavelength of 0.49986(1) Å, calibrated from the positions of 8 reflections from a LaB_6_ reference material. The low-temperature evolution of the crystal structure was also studied by neutron powder diffraction performed on the high-resolution powder diffractometer SPODI at the research reactor FRM-II (Garching, Germany) with monochromatic neutrons of 1.5481(1) Å wavelength.^27^ Measurements were performed in the Debye–Scherrer geometry. The powder sample was filled under an argon atmosphere into a thin-wall vanadium can and mounted in a top-loading closed-cycle refrigerator. Helium 4.6 was used as a heat transmitter. The instantaneous temperature was measured using two thin-film resistance cryogenic temperature sensors Cernox and controlled by a temperature controller from LakeShore. Two-dimensional powder diffraction data were collected at 4, 60, and 100 K, and then corrected for geometrical aberrations and curvature of the Debye–Scherrer rings. All diffraction patterns have been analyzed by full-profile Rietveld refinements, using the FullProf program with the user interface WinPLOTR.^28^ The atomic positions including oxygen were refined with an isotropic approximation for the thermal displacement parameters, which were refined independently for Cu, Re, and O in the case of neutron experiments and constrained to be equal for oxygen atoms for synchrotron experiments.

### 2.2 High-Pressure Synchrotron Single-Crystal and Powder Diffraction Studies

High-pressure single-crystal and powder diffraction measurements were performed in an angle-dispersive mode at the ID09A beamline of the ESRF (Grenoble) (λ = 0.4144 Å) at room temperature. High pressures were generated by means of the diamond anvil cell (DAC) technique. The samples were placed in spark-eroded holes of preindented metal (Re) gaskets, together with small ruby spheres for pressure determination and helium as pressure-transmitting medium. Pressure was measured before and after each data collection. In single-crystal experiments, the extraction and correction of the intensity data, merging of reflections, and refinements of the lattice parameters were done with the CrysAlis program (Agilent Technologies). The structure refinements were carried out with the SHELXL software^29^ integrated into the WingX system. Only the single-crystal data up to 1.5 GPa could be successfully integrated and used for structure refinement. At higher pressures, a structural phase transition led to rapid deterioration of the crystal quality. Therefore, the data measured on powder were employed instead and analyzed by the Le Bail profile fitting using FullProf with WinPLOTR.^28^

### 2.3 High-Pressure Raman Spectroscopy Studies

High-pressure Raman measurements were performed at room temperature with a four-pin type diamond anvil cell with 0.4 mm flat culets.^30^ The sample, together with a ruby chip for pressure calibration, was loaded into a 0.2 mm hole in the rhenium gasket with helium as the pressure-transmitting medium. Since CuReO_4_ is sensitive to moisture and for rhenium, an inert atmosphere is desired, the loading was performed in a glovebox under an argon atmosphere. The gasket dimensions in our experiments were 3 × 3 × 0.25 mm^3^.

The pressure was measured by using the ruby fluorescence technique. Raman spectra at pressures up to 7 GPa were collected using a LabRam spectrometer (NeHe excitation 15 mW laser with wavelength 632.8 nm, grating 1800, confocal hole 1100 μm, 50× objective).

### 2.4 Electronic Structure Calculations

Thermodynamics and electronic structures of the CuReO_4_ polymorphs were assessed using density-functional theory (DFT) band-structure calculations. Crystal structures were relaxed in VASP^31,32^ using the r^2^SCAN functional.^33^ For each phase, total energies were obtained at several constant volumes, with all atomic positions relaxed at each volume and fitted with the Murnaghan equation of state to evaluate the bulk modulus and calculate the enthalpy. Electronic band structures were calculated within a full-potential local-orbital code (FPLO)^34^ using the PBE exchange-correlation functional^35^ and within VASP using the hybrid functional HSE06.^36^ In both cases, experimental lattice parameters were used, whereas atomic positions were optimized in VASP (with r^2^SCAN) prior to the band-structure calculations.

## 3 Results and Discussion

### 3.1 Thermal Behavior of CuReO_4_ at Ambient Pressure

Our data show no evidence for temperature-induced phase transitions in CuReO_4_ at ambient pressure in the temperature range between 4 K and the melting point of the compound at 700 K. Examples of synchrotron powder diffraction patterns for CuReO_4_ with the Rietveld analysis of the diffraction data are shown in Figure S1 (Supporting Information) for three different temperatures. For Rietveld analysis, the room-temperature structural model from the work^20^ was applied.

**Figure 1:**
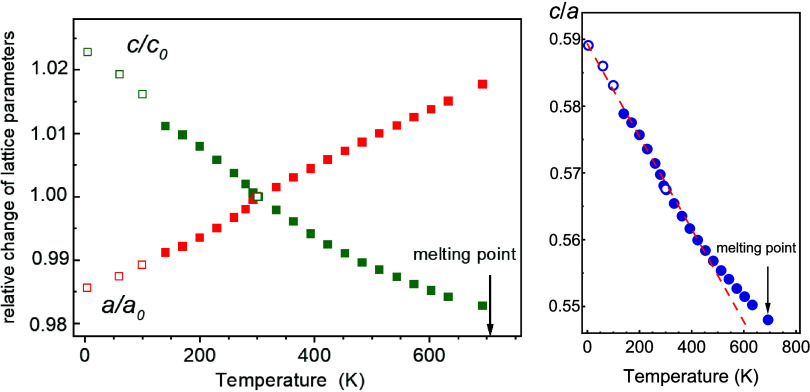
Relative changes of the lattice parameters vs temperature for the ambient pressure CuReO_4_–AP polymorph (left). The values were normalized to the lattice parameters at room temperature. Right: temperature dependence of the *c*/*a* ratio for CuReO_4_–AP together with the linear extrapolation of the data (red dashed line). Empty symbols correspond to the neutron powder diffraction data.

**Figure 2:**
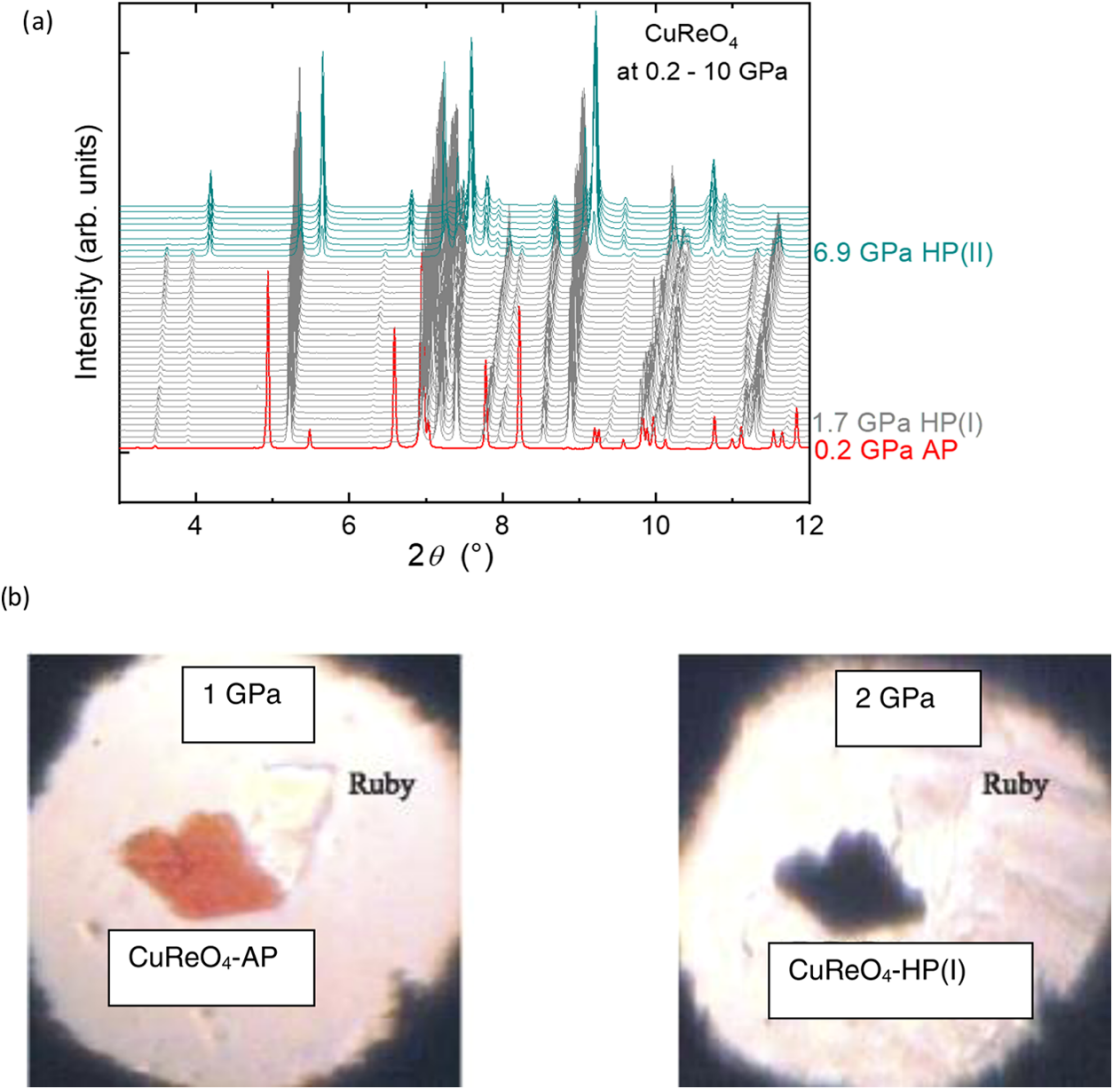
(a) Selected high-pressure synchrotron powder diffraction patterns collected between 0.2 and 10 GPa. (b) Color of the CuReO_4_ crystal before and after the first high-pressure phase transition at 1.5 GPa.

A positive thermal expansion along the *a*-axis with an average thermal expansion coefficient α_a_ = 4.90(6) × 10^–5^ K^–1^ and a negative expansion along the *c*-axis with α_c_ = – 6.00(21) × 10^–5^ K^–1^ were determined in the investigated temperature range from the combined analysis of synchrotron and neutron powder diffraction data, see Figure 1, left. Some deviation from the linear behavior of the *c*/*a* ratio with the temperature was detected above 400 K (Figure 2, right).

A negative thermal expansion is not uncommon in tetrahedrally coordinated structures, such as zinc-blende and wurtzite structures, or cuprite-like structures with bridging atoms having the coordination number CN = 2.^37^ Although some parameters seem to be essential for negative expansion (for example, a large charge separation and the ionic character of the chemical bond, or the strong M–O–M bridges), any model for a tension-driven mechanism should be considered individually for each solid.^37^ For example, copper(I) chloride, CuCl with the diamond-like structure and ionic bonding demonstrates negative thermal expansion, whereas in diamond with its covalent bonding, the expansion remains positive.^37^ Negative expansion over a broad temperature range was also observed for cuprite-like Ag_2_O, although the OAg_4_ tetrahedra distort and expand on heating due to increasing the average Ag–O bond length, while the average Ag–Ag nearest neighbor distance decreases, reflecting the negative bulk expansion.^37^ A stronger compressibility of the ionic bonds compared to the covalent bonds in complex compounds was discussed in ref (38).

Another explanation for the negative thermal expansion of CuReO_4_ along the *c*-axis and its nonlinearity with the temperature can relate to the chain-like character of the structure in this direction. In CuReO_4_, CuO_4_- and ReO_4_-tetrahedra build spirally twisted rings, or chains, composed of 4-, 6-, 8-, and 10-MeO_4_ polyhedra. The 10-fold chains form big channels along the *c*-axis,^20^ see also Figure 5b, left.

Theoretical studies of dynamic processes in chain-type structures with the temperature show that longitudinal and transverse vibrations of a chain, containing interacting species, lead to an entire negative thermal expansion at low temperatures and a positive expansion at high temperatures.^39^ The change from the negative to the positive expansion is not abrupt, resulting in a nonmonotonous temperature behavior.

Hence, we suppose that both, negative thermal expansion along the *c*-direction and observed deviation from the linearity of *c*/*c*_0_ and *c*/*a* above 400 K (Figure 1), are probably associated with competitive dynamic processes in MeO_4_-chains around channels in the nondense CuReO_4_ structure.

From neutron powder diffraction data, it was possible to precisely determine the coordinates of the light oxygen atoms in CuReO_4_. This way, the temperature evolution of the Cu–O and Re–O distances could be followed. Whereas the average Re–O distance remains nearly unchanged (1.739(7) Å at 300 K and 1.736(2) Å at 4 K), the average Cu–O bond increases from 2.015(7) Å at 300 K to 2.030(3) Å at 4 K. The distortion of both CuO_4_- and ReO_4_-polyhedra increases with temperature: the ratio ⟨M–O⟩_shortest_/⟨M–O⟩_longest_ is 0.900 (4 K) and 0.885 (300 K) for CuO_4_, while 0.982 (4 K) and 0.971 (300 K) for ReO_4_.

For a comparison of CuReO_4_ thermal behavior with other perrhenates, we studied silver perrhenate Ag^+^Re^7+^O_4_ with the tetragonal scheelite-type structure that features isolated ReO_4_-tetrahedra and Ag-atoms in oxygen dodecahedra, connected by edge-sharing. A powdered sample of AgReO_4_ was synthesized from Re_2_O_7_ and Ag_2_O at 773 K in an evacuated quartz tube. AgReO_4_ also shows an anisotropic expansion, but it is positive along both directions, with the linear thermal expansion coefficients of 2.55(4) × 10^–5^ K^–1^ along the *a*- and 4.33(10) × 10^–5^ K^–1^ along the *c*-axis over the temperature range of 100–713 K (see Figure S2 of the Supporting Information). Below 50 K and down to at least 21 K, the expansion becomes negative for both crystallographic directions, *a* and *c*. It is worth noting that the perrhenates with the scheelite structure usually show positive thermal expansion along both *a* and *c*.^40^

### 3.2 High-Pressure Behavior of CuReO_4_ at Room Temperature

#### 3.2.1 Structural Phase Transitions

The negative thermal expansion of the Cu–O bonds in CuReO_4_ can be a driving force for pressure-induced phase transition(s). Indeed, our high-pressure experiments clearly show drastic changes in the diffraction patterns above 1.5 GPa [HP(I) field] and above about 7 GPa [HP(II) field] in comparison to the ambient pressure pattern; see Figure 2a. A change in the sample color from orange to black was observed above 1.5 GPa (Figure 2b).

**Figure 3:**
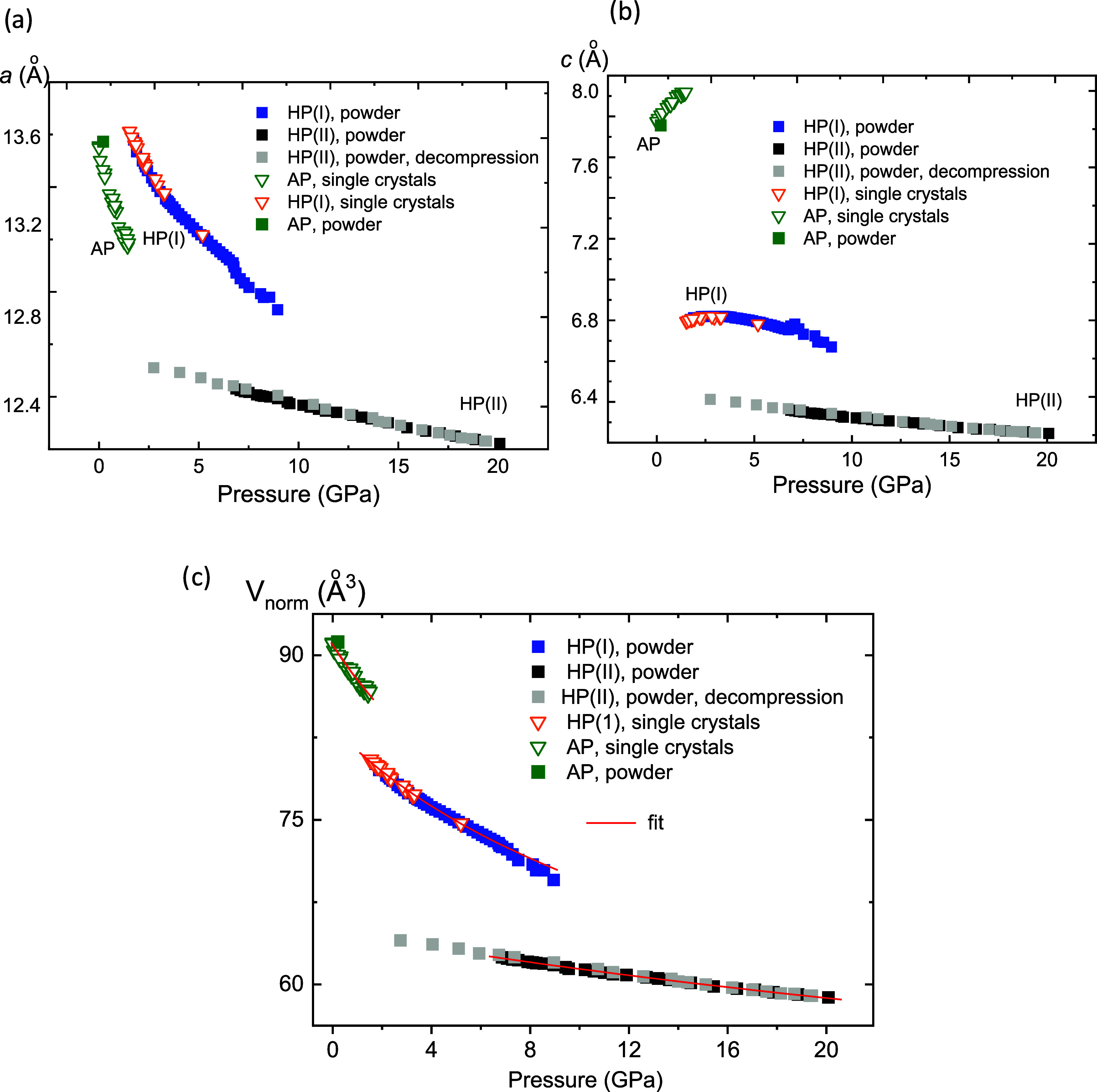
Pressure dependence of lattice parameters *a* (a) and *c* (b) and the cell volume per formula unit (c). The data were obtained by the single-crystal (triangles) and powder (squares) synchrotron diffraction experiments upon compression. The gray squares correspond to the decompression of the second high-pressure polymorph CuReO_4_–HP(II). Solid lines are the fits with the Murnaghan equation of state with the fixed *B*_0_′ = 4.4.^50^

All diffraction patterns measured up to 20 GPa could be indexed by using the *I*-centered tetragonal unit cell. Between ambient pressure and 1.5 GPa, within the AP phase, the *c*-axis is slightly expanded from 7.7729(1) to 7.9187(1) Å, whereas the *a*-axis is shortened from 13.6965(2) to 13.2333(2) Å, see Figure 3. This behavior is consistent with the temperature evolution of the CuReO_4_ lattice upon cooling at ambient pressure. The lattice contraction corresponds to the coefficients α_a_ = 22.5(9) × 10^–3^ GPa^–1^ and α_c_ = – 1.27(5) × 10^–3^ GPa^–1^ between ambient pressure and 1.5 GPa.

According to the single-crystal diffraction experiments, the average Cu–O (2.030(8) Å) and Re–O (1.720(9) Å) bond lengths remain nearly the same up to 1.5 GPa. The volume decrease is accomplished mainly by tilting the CuO_4_ and ReO_4_ tetrahedra.

Above 1.5 GPa, a significant reduction in the cell volume, a drastic increase in the *a*-parameter, and a decrease in the *c*-parameter were observed, indicating a formation of the first high-pressure polymorph CuReO_4_–HP(I), see Figure 3. The pressure evolution of the lattice parameters for CuReO_4_–HP(I) exhibits an anomaly, with the *c*-parameter showing a weak but well-defined maximum around 4 GPa. Calculation of the lattice contraction results in the coefficients α_a_ = 8.70(2) × 10^–3^ GPa^–1^ between 1.52 and 6.72 GPa, while along the *c*-direction, the lattice first expands within 1.52–2.4 GPa (α_c_ = – 2.4(2) × 10^–2^ GPa^–1^) and then contracts between 4.0 and 6.72 GPa with α_c_ = 2.18(6) × 10^–2^ GPa^–1^.

The formation of the second high-pressure polymorph CuReO_4_–HP(II) above 7 GPa was accompanied by abrupt shortening of both the *a*- and *c*-parameters (Figure 3). The second polymorph is stable at least up to 20 GPa. Pressure-induced lattice contraction is described by α_a_ = 1.58(2) × 10^–3^ GPa^–1^ and α_c_ = 1.37(3) × 10^–3^ GPa^–1^ between 6.86 and 20.08 GPa.

Only a limited information on the high-pressure structural behavior of perrhenates is presently available. Among the perrhenates with a monovalent cation, CuReO_4_ is the only example of preserving tetragonal lattice symmetry across two pressure-induced phase transitions despite the very large volume changes Δ*V*/*V*_0_ of −7.2% for CuReO_4_–HP(I) and −14.3% for CuReO_4_–HP(II). These volume changes are larger compared to the scheelite-type orthorhombic TlReO_4_ that transforms into another orthorhombic structure with zero volume change at 1 GPa, into a monoclinic wolframite-type structure at 2 GPa with a Δ*V*/*V*_0_ of −2%, and further into a monoclinic BaWO_4_–II-type structure above 10 GPa with Δ*V*/*V*_0_ of −9%.^14^

#### 3.2.2 Crystal Structures of the High-Pressure Polymorphs

**Figure 4:**
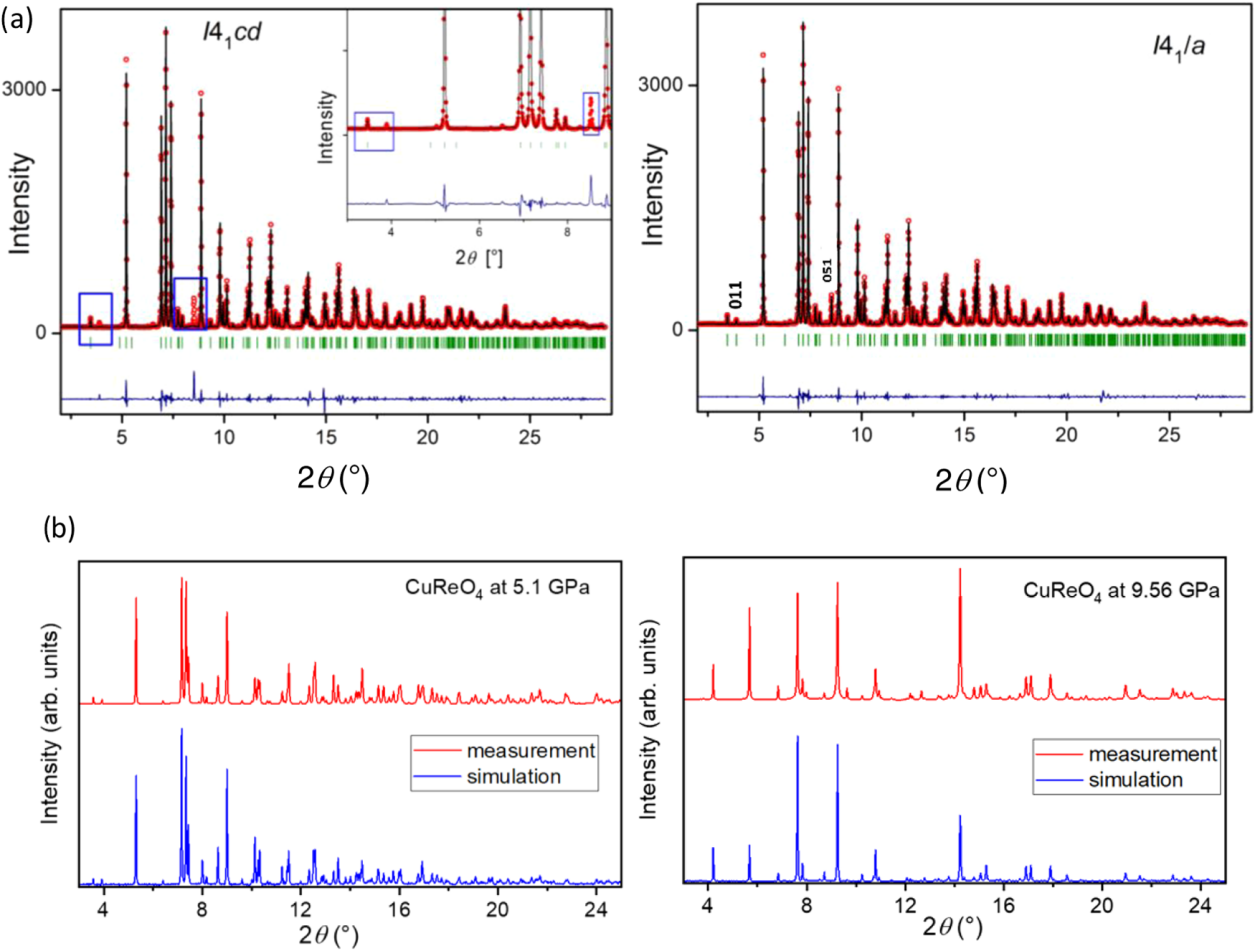
(a) Diffraction patterns of CuReO_4_–HP(I) at 1.73 GPa with the experimental (red), theoretical (black, Le Bail refinement), and differential (blue) curves, analyzed using *I*4_1_*cd* and *I*4_1_/*a* space groups. Two notable Bragg reflections, 011 and 051 at 2θ = 3.9 and 8.7°, respectively, are inconsistent with the *I*4_1_*cd* space group (left) and indicate the *I*4_1_/*a* space group (right). (b) Experimental (red) and calculated (blue) diffraction patterns of CuReO_4_–HP(I) at 5.1 GPa and of CuReO_4_–HP(II) at 9.56 GPa. The calculated curves are based on the atomic positions obtained from DFT.

Above 1.5 GPa, a major structural transformation toward the first high-pressure polymorph CuReO_4_–HP(I) occurs. Although the unit cell remains body-centered tetragonal, the transition is not symmetry conserving (not isosymmetric) because both single-crystal and powder data show two strong Bragg reflections, 001 and 051, that violate the *I*4_1_*cd* symmetry of the AP polymorph (Figure 4a). The single-crystal data between 1.5 and 5 GPa give strong evidence of the centrosymmetric *I*4_1_/*a* space group of CuReO_4_–HP(I). A direct structure refinement was not possible because crystals crushed upon the transition due to the large volume difference between the AP and HP(I) polymorphs. It was also difficult to achieve a complete structure refinement using powder data because of preferred orientation effects and the weak scattering from oxygen in the presence of heavy Re atoms. Therefore, we used a combined approach where Cu and Re were tentatively located using the XRD data, while oxygen positions were obtained from the structure optimization in DFT. The complete structural model is given in Table 1. Figure 4a shows excellent agreement between the experimental XRD pattern and the pattern simulated using this structural model.

**Table 1:** Atomic Positions of CuReO_4_–HP(I) at 5.1 GPa (*I*4_1_/*a*, *Z* = 16, *a* = 13.2738 Å, *c* = 6.7954 Å, *V* = 1197.31 Å^3^), Determined by DFT Structure Relaxation, together with the Average Cu–O and Re–O Distances in the CuO_4_ Tetrahedra and ReO_6_ Octahedra

atom	site	*x*	*y*	*z*	*d*(*M*–O), Å
Re_1_	16*f*	0.84631	0.11062	0.39356	1.9317
Cu_1_	16*f*	0.88499	0.87585	0.64126	1.9354
O_1_	16*f*	0.31764	0.41171	0.38199	
O_2_	16*f*	0.32939	0.24016	0.13277	
O_3_	16*f*	0.84188	0.94655	0.40005	
O_4_	16*f*	0.02289	0.89175	0.57485	

**Figure 5:**
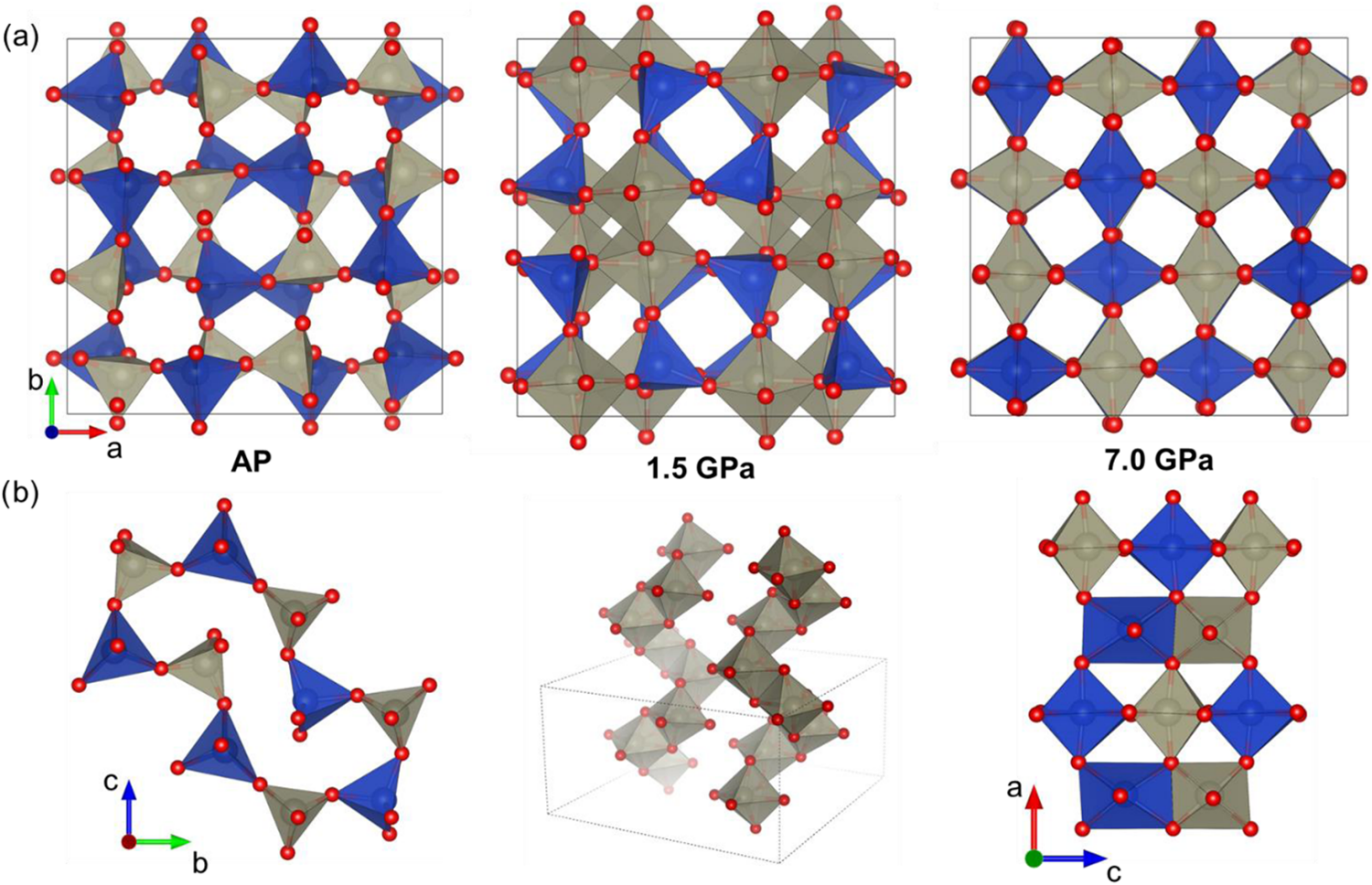
(a) Crystal structures of three CuReO_4_ polymorphs in the *ab*-plane. The Re and Cu polyhedra are gray and blue, respectively. The Cu and Re atoms change their oxygen surroundings from tetrahedra (ambient pressure) to distorted octahedra (7.0 GPa). (b) From left to right: 10-membered rings of the CuO_4_- and ReO_4_-tetrahedra in pristine CuReO_4_–AP (left), forming the side of the channels along the *c*-axis; a structural fragment of CuReO_4_–HP(I) with infinite chains of the edge-sharing ReO_6_-octahedra (middle), and a fragment of CuReO_4_–HP(II) with the edge-sharing ReO_6_/CuO_6_-octahedra.

The main changes in the CuReO_4_–HP(I) structure compared to those of the AP polymorph include the transformation of the ReO_4_ tetrahedra into distorted ReO_6_ octahedra with four short Re–O bonds of 1.74–1.85 Å and two long Re–O distances of 2.18–2.20 Å. These octahedra share common edges and form infinite chains along the *c*-axis, thus reducing the size of the channels along this axis (Figure 5). With these chains of the ReO_6_-polyhedra, the structure somewhat resembles the wolframite-like structure of CuWO_4_ that has chains of the edge-sharing WO_6_-octahedra. However, the CuO_4_-polyhedra in CuReO_4_–HP(I) are still not connected to each other, in contrast to the infinite chains of the edge-sharing distorted CuO_6_-octahedra in CuWO_4_.^41^ The Cu–O bond lengths in CuReO_4_–HP(I) lie in the range 1.90–1.97 Å, whereas the fifth oxygen atom is at 3.06 Å away from Cu.

The phase transition to CuReO_4_–HP(I) can be classified as a pseudoreconstructive, similar to the high-pressure phase transition in the K_2_Co_2_Mo_3_O_12_ polyanionic structure.^41^ On the one hand, the connectivity of all atomic sites in these two structures is not broken, as typical for a displacive phase transition. On the other hand, two new Re–O bonds are formed (typical for a reconstructive phase transition), resulting in the infinite chains of the edge-sharing ReO_6_-octahedra. In K_2_Co_2_Mo_3_O_12_, the MoO_4_-tetrahedra transform under pressure into MoO_5_-pyramids and MoO_6_-octahedra.^42^

The first high-pressure transition is fully reversible and shows only a small pressure hysteresis. After decompression from 5 GPa, all features of the ambient-pressure CuReO_4_–AP form were restored, and the samples color turned back from black to red.

Despite the denser nature of the CuReO_4_–HP(I) structure, it still contains channels along the *c*-axis, making possible further transformations upon increasing pressure.

Above 6.7 GPa, a second CuReO_4_–HP(II) polymorph started to form. Its powder diffraction pattern is consistent with the *I*4_1_/*a* symmetry as well, suggesting that the HP(I)–HP(II) transition is symmetry conserving (isosymmetric). A coexistence region of the two phases, CuReO_4_–HP(I) and CuReO_4_–HP(II), between 6.72 and 8.10 GPa, points to a certain kinetic barrier upon the transformation. The volume drop Δ*V*/*V*_0_ of −14.3% for the CuReO_4_–HP(I) → CuReO_4_–HP(II) transition is twice larger than for the CuReO_4_–AP → CuReO_4_–HP(I) transition. During decompression, only the CuReO_4_–HP(II) phase was observed down to 2 GPa, followed by an amorphization of the sample upon further pressure release.

We surmised that CuReO_4_–HP(II) crystallizes in an ordered rutile-like structure of the NbO_2_-type^43^ and confirmed this conjecture using DFT calculations, see Table 2 for the structural model and Figure 4b for a comparison between the experimental and simulated XRD patterns. The Cu and Re atoms occupy the Nb(1) and Nb(2) positions in the oxygen octahedra with longer (Cu) and shorter (Re) bonds to the oxygen atoms. Both CuO_6_- and ReO_6_-octahedra are strongly distorted, with the metal–oxygen distances varying between 1.97–2.26 Å for Cu and 1.84–1.99 Å for Re, similar to the NbO_6_-polyhedra in the NbO_2_-structure. Calculated changes in metal–oxygen distances and metal–oxygen polyhedra, dependent on the pressure, are shown in Figure S3 of the Supporting Information.

**Table 2:** Atomic Positions of CuReO_4_–HP(II) at 9.56 GPa (*I*4_1_/*a*, *Z* = 16, *a* = 12.4670 Å, *c* = 6.3254 Å, *V* = 983.13 Å^3^), Determined by DFT Structure Relaxation, together with the Average Cu–O and Re–O Distances in the CuO_6_- and ReO_6_-Octahedra

atom	site	*x*	*y*	*z*	*d*(*M*–O), Å
Re_1_	16*f*	0.62789	0.87049	0.26072	1.8916
Cu_1_	16*f*	0.13247	0.37182	0.26735	2.0508
O_1_	16*f*	0.12724	0.22175	0.27463	
O_2_	16*f*	0.12078	0.22352	0.73135	
O_3_	16*f*	0.12747	0.52107	0.29347	
O_4_	16*f*	0.61993	0.02363	0.23891	

Owing to the similarity between the CuReO_4_ ambient-pressure structure and the structures of some silicon dioxides and aluminosilicates with spirally twisted rings of the MO_4_-tetrahedra (M = Si and Al),^20^ the pressure-induced transformation toward the rutile-like structure is not surprising, since SiO_2_ and aluminosilicates are known to transform into rutile-type structures.^23^ It is more surprising that CuReO_4_–HP(I) exhibits similarities to the CuWO_4_ wolframite-like structure, but in contrast to this structure, no infinite chains of the CuO_6_-octahedra form. The difference in the valence states of the cations between Cu^1+^Re^7+^O_4_ and Cu^2+^W^6+^O_4_ probably plays a role here. The tetrahedral coordination of Cu in the HP(I) polymorph indicates that Cu most likely remains in the 1+ state, as confirmed by the DFT calculations below. Moreover, even in the HP(II) polymorph, the CuO_6_-octahedra does not show the Jahn–Teller distortion that would be typical for Cu^2+^. On the other hand, the Jahn–Teller distortion of the Cu^2+^O_6_-octahedra may be significantly alleviated under pressure, as in CuWO_4_ that shows an increase in the crystal symmetry under pressure.^44^

#### 3.2.3 High-Pressure Raman Spectroscopy

**Figure 6:**
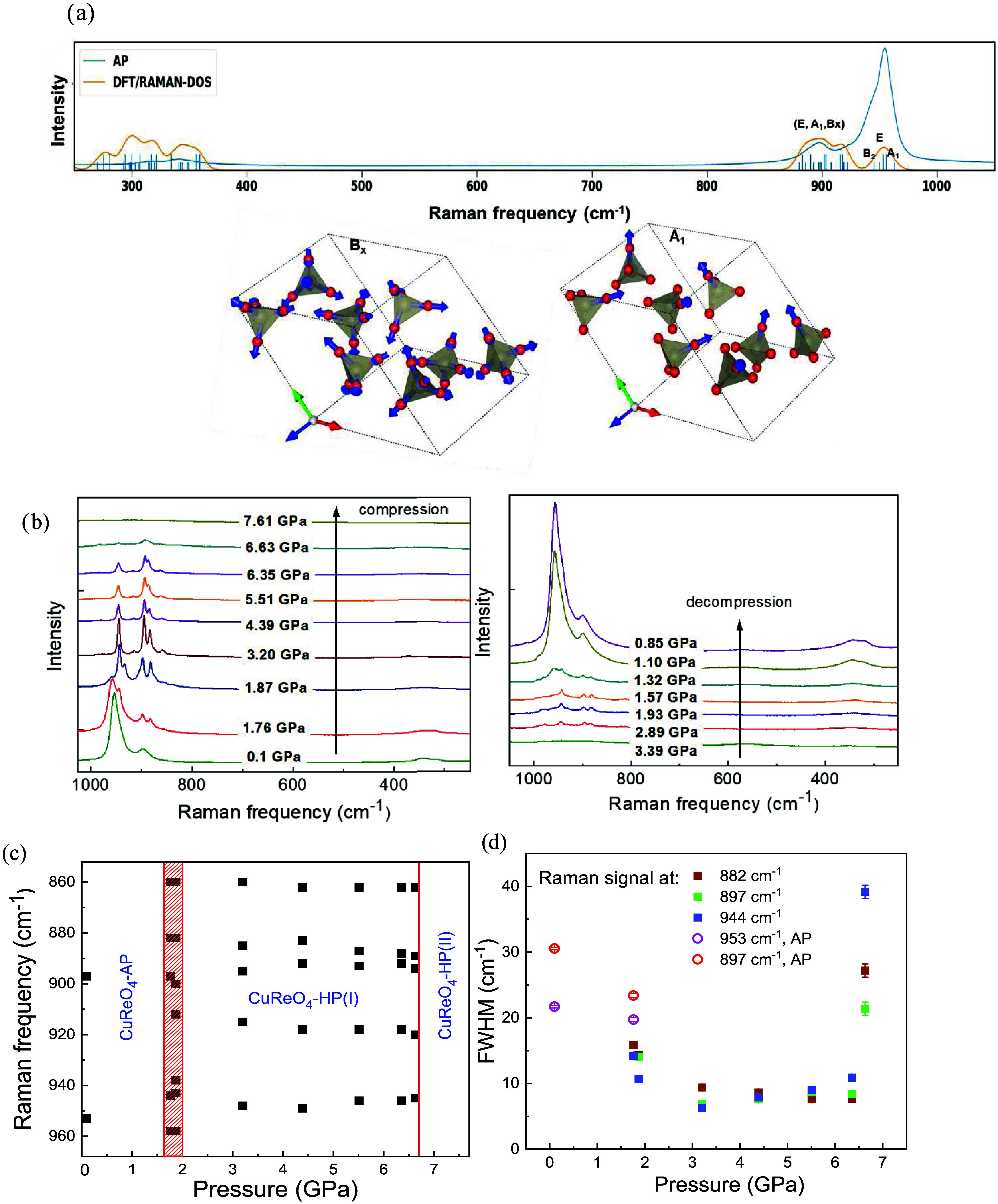
(a) Raman spectrum of CuReO_4_–AP compared to the DFT phonon density of states at the Γ-point. The lower panel shows displacement vector orientation for representative modes, such as symmetric and asymmetric stretching vibration of Re–O bonds. (b) Raman spectra of CuReO_4_, collected during compression (left) and decompression (right). The spectra are shifted along the intensity axis for better clarity. (c). Pressure dependence of the intense high-frequency Raman signals of CuReO_4_ during compression. The red vertical line/area marks the phase transitions. The CuReO_4_–AP and CuReO_4_–HP(I) polymorphs coexist in the narrow pressure range of about 1.7–1.9 GPa. (d) The full width at half-maximum (FWHM) of intense high-frequency Raman signals of CuReO_4_–HP(I) in dependence on the pressure, together with CuReO_4_–AP. A peak broadening and intensity decrease of Raman signals above 6.6 GPa point to the collapse of the CuReO_4_–HP(I) structure.

The pressure-induced phase transitions in CuReO_4_ can also be followed by Raman spectroscopy. Room-temperature Raman spectra obtained during compression and decompression are shown in Figure 6. For monovalent perrhenates, there is a clear separation of the vibrational modes of the tetrahedral ReO_4_^–^-group from the low-frequency external lattice vibrational modes.^12,15,18,45,46^ For example, in alkali perrhenates AReO_4_ (A = Na–Cs), AgReO_4_, and TlReO_4_ with the tetragonal scheelite or orthorhombic pseudoscheelite structures,^46^ and in LiReO_4_ with the ZnMoO_4_ structure type,^47^ the lattice modes were observed in the 40–120 cm^–1^ range, while the internal modes of ReO_4_^–^ (both symmetrical and asymmetrical) can be divided into two groups, at 320–350 cm^–1^ (bending modes) and 900–970 cm^–1^ (symmetric and antisymmetric stretching modes). The frequency of the symmetric Re–O vibration ν(ReO_4_^–^) at about 900 cm^–1^ depends not only on the cationic radius and crystal structure but also on the polarization effect of the cation: while ν(ReO_4_^–^) increases linearly with increasing the alkali-metal cation radius, the monovalent Ag^+^ and Tl^+^ do not follow this linear trend.^46^

Figure 6a represents a detailed analysis of the vibrational spectrum for the ambient-pressure phase. With the space group *I*4_1_*cd*, it upholds the point symmetry group *C*_4*v*_ (4 mm) and the 144 phonon modes at the Γ-point have the following irreducible representations: Γ(*C*_4*v*_) = 18A_1_(R) + 18A_2_(IR) + 18B_1_(R) + 18B_2_(R) + 72E(R), where the A_2_ mode is IR-active for the respective symmetry. DFT/PAW theory was employed at the PBE level to evaluate phonon modes in the γ point.^31,32,48,49^ The atomic positions were refined at the PBE level with an accuracy of 10^–8^ eV, using a Γ-centered *k*-grid of 2 × 2 × 3, while unit cell parameters were kept at the r2SCAN level. The complete set of Γ-point phonon modes, including their energies and assigned symmetry assessments, is summarized in Table S1 of the Supporting Information.

In Figure 6a, phonons and their densities of states are weighted by multiplicity, as computing Raman intensities lie beyond the scope of this research. Overall, the structure and position of high- and low-frequency phonon mode bundles are predicted satisfactorily well. It emphasizes that experimentally observed lines do not represent a distinct signal but rather a combination of multiple modes.

Table 3 summarizes the high-frequency part of the spectrum for CuReO_4_–AP with an assessment of the experimentally observed features. Here, the higher frequency components are mostly dominated by stretching vibrations occurring at around 950 cm^–1^.

**Table 3:** DFT-Computed and Experimental Vibrational Frequencies of Ambient-Pressure CuReO_4_ and Description of Vibrational Modes^a^

CuReO_4_–AP, *I*4_1_*cd*			
symmetry	DFT results	exp. Raman signals	mode^b^
	856–894	897(br)	δ(Re–O), mult
B_2_(137)	920	942(sh)	ν(Re–O)
B_1_(138)	925		ν(Re–O)
E(139,140)	927	955(vs)	ν(Re–O), asym
E(141,142)	930		ν(Re–O), asym
A_1_(143)	935		ν(Re–O), sym

a Vibrational frequencies are in cm^–1^, and relative intensities are “vs”—very strong, “s”—strong, “sh”—shoulder, “br”—broad.

b The mode description highlights the main type of contributing coordinates, but the overall mode composition is usually more complex. Abbreviations: δ(Re–O)—deformational mode, including small changes of the valence angles; ν(Re–O)—symmetric and asymmetric stretching vibration of Re–O bonds; “mult”—multiple δ(Re–O) deformation modes including breathing mode of ReO_*x*_ polyhedra.

The most significant changes in the CuReO_4_ spectra upon increasing pressure are visible in the ReO_4_-internal modes region at 880–950 cm^–1^ between 0.1 and 3.2 GPa during compression and decompression (Figure 6b). The spectrum with a splitting of peaks at 1.76 GPa during compression can be understood as a superposition of two CuReO_4_ polymorphs, CuReO_4_–AP and CuReO_4_–HP(I). The separation of the symmetric and asymmetric stretching modes ν at about 955 cm^–1^ suggests different lengths of the Re–O bonds of these two polymorphs because the vibration frequency is dependent on the Re–O bond lengths.^15^ At higher pressures, the almost constant frequency of ν(ReO_4_^–^) = 954 cm^–1^ indicates very weak compressibility of the ReO_*x*_-polyhedra in the HP(I) polymorph. A similar incompressibility of the ReO_4_-tetrahedra under pressure was observed in the scheelite-like AgReO_4_.^15^

From 1.76 to 1.87 GPa, the amount of CuReO_4_–AP is reduced, as seen in the Raman spectrum. The coexistence of these two phases and the abrupt change in the unit-cell volume suggest the first-order character of the phase transition between the AP and HP(I) polymorphs. The significant difference between the FWHM values of Raman signals for CuReO_4_–AP and CuReO_4_–HP(I) also confirms changes in the structure symmetry and/or in the oxygen surrounding of rhenium cations (Figure 6d).

Between 3.2 and ca. 7 GPa, the spectra show features of the HP(I) polymorph only, in agreement with the high-pressure synchrotron diffraction data. The fwhm values of the high-frequency Raman signals are nearly constant in this region.

Theoretical analysis of a CuReO_4_–HP(I) spectrum is presented in Figure S4 and Table S2 of the Supporting Information. Above 6.6 GPa, a collapse of the CuReO_4_–HP(I) structure is concluded due to a huge peak broadening and intensity decrease of Raman signals. In the stability region of the HP(II) polymorph above 7 GPa, Raman signals vanished completely (Figure 6b).

The presence of the crystalline phase above 7 GPa witnessed by the diffraction data together with the absent Raman signal indicates the likely metallization of CuReO_4_, which is confirmed by the DFT calculations below.

All changes in the Raman spectra are reversible, although some hysteresis is observed during pressure release (Figure 6b). This confirms the proposed first-order character of the first-phase transition. The transition pressures for AP → HP(I), determined with high-pressure synchrotron diffraction and laboratory Raman spectroscopy experiments, are satisfactorily matched. Coexistence of both polymorphs in Raman experiments up to 1.87 GPa, which was not observed in diffraction measurements, can be caused by a slight inhomogeneity during compression.

### 3.3 Structural Stability and Electronic Structure

**Figure 7:**
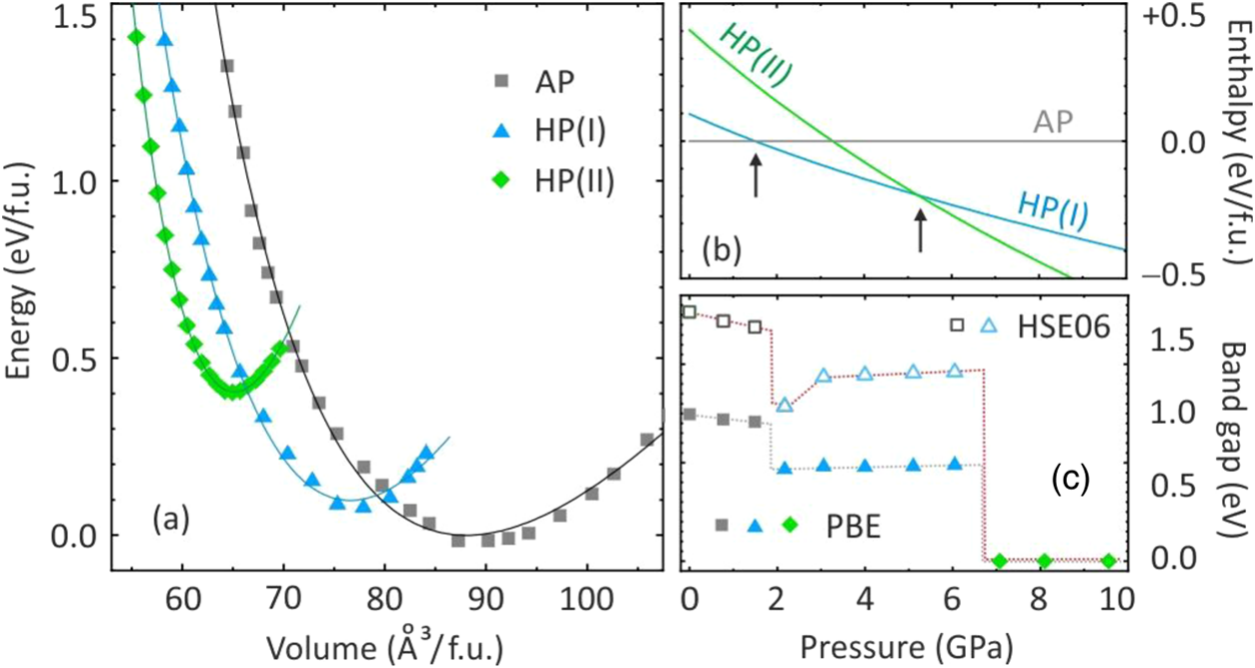
(a) Total energies of three CuReO_4_ polymorphs calculated at fixed unit-cell volumes. The lines are the fits to the Murnaghan equation of state. (b) Calculated enthalpies given relative to the enthalpy of the AP polymorph. The arrows show the transitions between the different polymorphs. (c) Electronic band gaps of the CuReO_4_ polymorphs calculated using the PBE (filled symbols) and HSE06 (empty symbols) functionals. The lines are guide-for-the-eye only.

Here, we verify the sequence of structural phase transitions by analyzing the thermodynamics of the CuReO_4_ polymorphs. Figure 7 shows the total energies of three CuReO_4_ polymorphs calculated at several fixed volumes. For fitting these energy-vs-volume curves, the Murnaghan equation of state was chosen; the parameters are listed in Table 4. The equilibrium volume decreases on going from CuReO_4_–AP to CuReO_4_–HP(I) and CuReO_4_–HP(II), whereas the equilibrium energy increases. The calculated bulk moduli (*B*_0_) are in reasonable agreement with the experimental values (Table 4). The increase in *B*_0_ from AP and HP(I) to HP(II) reflects the change in the connectivity of the structure from corner-sharing to edge-sharing of the Cu and Re polyhedra. This change is achieved by increasing the coordination number of Cu and Re from 4 to 6.

**Table 4:** Parameters of the Equation of State for the CuReO_4_ Polymorphs^a^

CuReO_4_	*E*_0_ (eV/fu)	*V*_0_ (Å^3^/fu)	*B*_0_ (GPa)	*B*_0_′	*V*_0_ (Å^3^/fu) exp.	*B*_0_ (GPa) exp.
AP	0	88.1(2)	32(2)	5.3(3)	90.9(1)	26(1)
HP(I)	0.10(1)	76.6(2)	57(4)	4.4(4)	83.45(6)	36.1(4)
HP(II)	0.40(1)	65.14(2)	152(2)	6.0(1)	64.86(4)	162(2)

a Equilibrium energy (*E*_0_), equilibrium volume (*V*_0_), bulk modulus at ambient pressure (*B*_0_) and pressure derivative of the bulk modulus (*B*_0_′), from DFT calculations, together with the experimental *B*_0_ and *V*_0_ values obtained by fitting pressure dependence of the unit-cell volume using the Murnaghan equation of state with the fixed *B*_0_′ = 4.4.^50^ The energies are given relative to the energy minimum of the AP polymorph.

Using the calculated enthalpies, we estimated the pressure of the AP-to-HP(I) transition as 1.5 GPa in perfect agreement with the experiment (Figure 7b). The HP(II) polymorph becomes thermodynamically stable above 5.3 GPa, whereas experimentally, it appears above 6.7 GPa with a broad pressure hysteresis. This hysteresis indicates that the transition is kinetically hindered and may, thus, be shifted toward higher pressures compared to the thermodynamic estimate.

**Figure 8:**
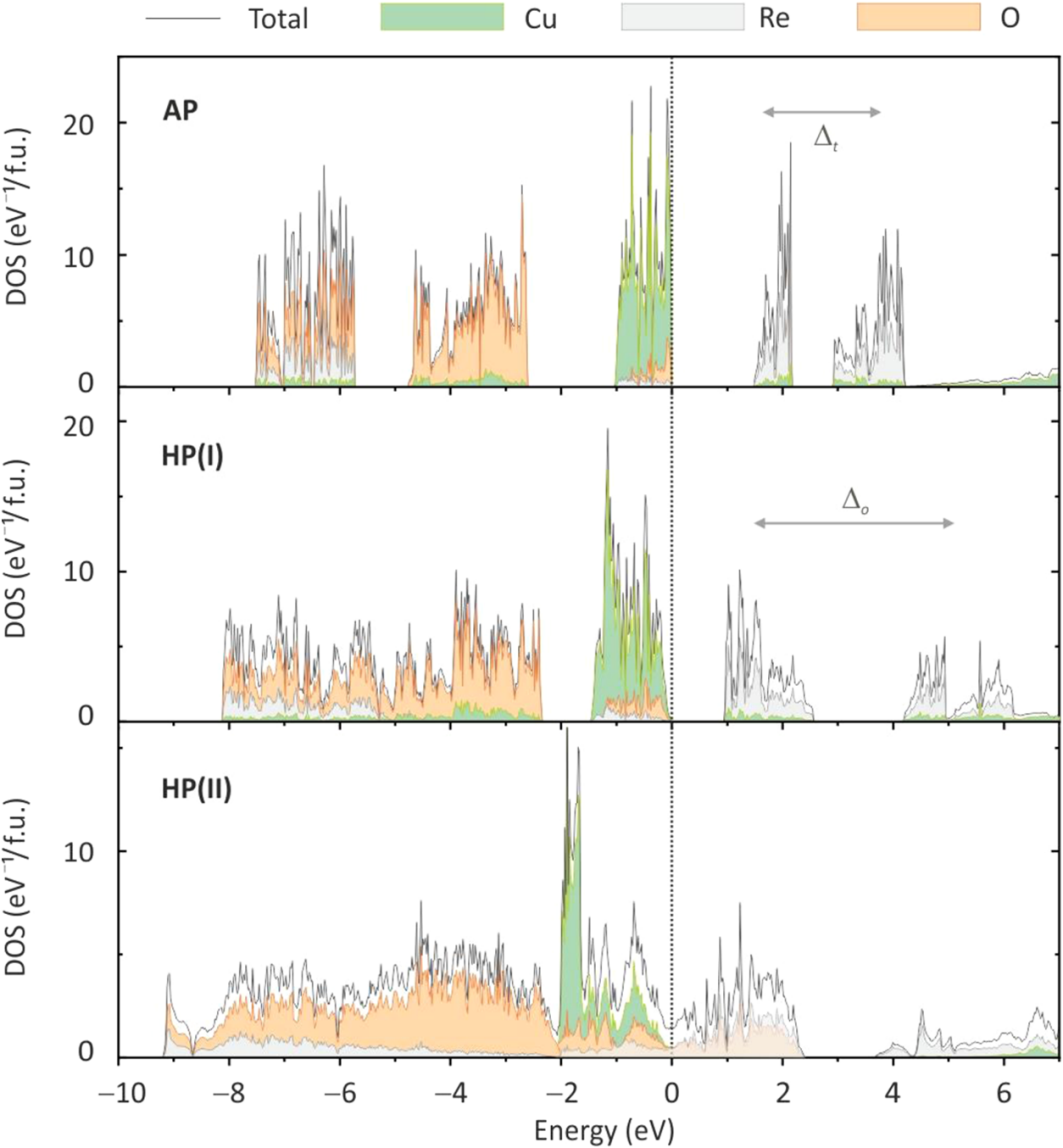
Electronic density of states for the CuReO_4_ polymorphs calculated using the PBE functional: AP at ∼0 GPa, HP(I) at 5.1 GPa, and HP(II) at 9.5 GPa. The Fermi level is at zero energy.

Figure 8 compares the electronic density of states for the CuReO_4_ polymorphs. The AP polymorph reveals two distinct oxygen bands, which are typical for compounds with tetrahedral polyanions.^51−53^ The lower-lying oxygen states arise from the bonding orbitals of the ReO_4_-tetrahedra and show a sizable contribution from Re 5d driven by the Re–O hybridization. The highest occupied states are predominantly Cu 3d, whereas Re 5d states are mostly found above the Fermi level and show *e*-*t*_2_ crystal-field splitting of the tetrahedrally coordinated transition metal atom. The splitting between the Cu 3d and Re 5d states gives rise to the band gap of 1.5–2.5 eV in the AP-polymorph (depending on the functional) that slightly decreases upon compression (Figure 7c). The mechanism of this reduction is probably similar to the one in AgReO_4_.^54^

Two oxygen bands merge in the HP(I) polymorph because the coordination of Re changes from tetrahedral to octahedral. This structural change also leads to a swap of the two Re bands above the Fermi level and increases their splitting because the octahedral crystal field is stronger than the tetrahedral one for the same anion. Consequently, the Re t_2g_ bands become lower in energy than the Re *e* bands in CuReO_4_-AP, and the band gap decreases by 20–30% in the HP(I) polymorph (Figure 7c).

The transformation into the HP(II) polymorph broadens both Cu 3d and Re 5d bands, resulting in their merging and the overall metallic behavior with the density of states of about 1.4 eV^–1^/fu at the Fermi level. These states feature almost equal contributions of Cu 3d, Re 5d, and O 2p. Importantly, most of the Cu 3d states remain below the Fermi level, suggesting that the Cu 3d shell is almost fully filled and only a minor charge transfer between Cu and Re happens under pressure. It means that the copper perrhenate should be described as Cu^1+^Re^7+^O_4_ within all three polymorphs. No valence state transition occurs in this compound within the pressure range of our study.

## 4 Summary and Conclusions

The presence of large channels in the ambient-pressure structure of CuReO_4_ facilitates structural transformations at moderate external pressures. Our XRD and Raman data consistently show two structural phase transitions below 10 GPa. The first transition at ∼1.5 GPa is accompanied by a change in the lattice symmetry from *I*4_1_*cd* to *I*4_1_/*a* and involves the tilt of the polyhedra along with the increase in the coordination number of rhenium from 4 to 6. In this HP(I) structure, the ReO_6_-octahedra are edge-sharing, building infinite chains that resemble the WO_6_-chains in the wolframite-like CuWO_4_ structure. Similar phase transitions, involving the rearrangement of a small number of the bonds of a single coordination polyhedron, are not uncommon in a wide variety of structure types and mostly involve a change in the space-group symmetry, for example, refs (55−57).

The first high-pressure transition in CuReO_4_ is of the first order according to the volume collapse of around 7%. Upon transition, the discontinuous shortening in the lattice parameter *c* and the significant expansion in the lattice parameter *a* is observed. It can be compared with the pressure-induced transitions in TlReO_4_, where the first orthorhombic scheelite-type high-pressure polymorph is stabilized already at around 1 GPa and features a tilting of the coordination polyhedra without any symmetry change.^14^ The second high-pressure TlReO_4_ polymorph forming around 2 GPa adopts a wolframite-like cell with an increased coordination number of Re from 4 to 6 and transforms further into a monoclinic BaWO_4_-type structure at even higher pressures. In CuReO_4_, an increase in the coordination numbers of Cu from 4 to 6 is observed in the second, rutile-like NbO_2_-type high-pressure phase above 7 GPa. This transition is first order as well. Interestingly, CuReO_4_ does not show any phase transitions at ambient pressure, in contrast to TlReO_4_ with its peculiar increase in symmetry upon cooling.^14,18,19^

The band gap of CuReO_4_ decreased under pressure. This trend is visible already from the color change above 1.5 GPa (Figure 2b) and confirmed by our DFT calculations. Intriguingly, the Raman signal vanishes in the HP(II) polymorph, indicating the likely metallic nature of this phase, in agreement with the DFT results. We thus conclude that CuReO_4_ becomes metallic already at 7 GPa, in contrast to other perrhenates that clearly show the Raman signal up to at least 15 GPa in TlReO_4_^12^ and 18 GPa in AgReO_4_,^15^ while no conclusive evidence for metallization has been obtained even at higher pressures. Interestingly, metalization occurs without any significant charge transfer between Cu^1+^ and Re^7+^. It is driven merely by the increased bandwidth that results in an overlap of the Cu 3d and Re 5d bands within the HP(II) polymorph. Our results further show that the rutile-like NbO_2_-type structure can be stabilized in perrhenates and in ternary oxides in general.

## Supporting Information Available

Additional diffraction data, structural data, and Raman data analysis (PDF)
