# Supplementary material for: Metallization without Charge Transfer in CuReO4 Perrhenate under Pressure
Source: Inorg Chem. 2025 Mar 20;64(12):6010–22. doi: 10.1021/acs.inorgchem.4c05051 (PMC11962830; doi:10.1021/acs.inorgchem.4c05051)
Supplement: Supplementary file 1 — ic4c05051_si_001.pdf [file ic4c05051_si_001.pdf]

Supporting Information

Metallization without charge transfer in $CuReO_4$ perrhenate under pressure

Daria Mikhailova[1*], Stanislav M. Avdoshenko[1], Maxim Avdeev[2,3], Michael Hanfland[4], Ulrich Schwarz[5], Yurii Prots[5], Angelina Sarapulova[6,7], Konstantin Glazyrin[8,9], Leonid Dubrovinsky[8], Anatoliy Senyshyn[10], Jens Engel[11#], Helmut Ehrenberg[12], Alexander A. Tsirlin[13, 14]

[1]*Leibniz Institute for Solid State and Materials Research Dresden (IFW Dresden), Helmholtzstr. 20, D-01069 Dresden, Germany*
[2]*Australian Nuclear Science and Technology Organisation, Lucas Heights, NSW 2234, Australia*
[3]*School of Chemistry, The University of Sydney, Sydney, NSW 2006, Australia*
[4]*European Synchrotron Radiation Facility, 71 Av. des Martyrs, 38000 Grenoble, France*
[5]*Max Planck Institute for Chemical Physics of Solids, Nöthnitzer Str. 40, D-01187 Dresden, Germany*
[6]*Freiburg Materials Research Center (FMF), Stefan-Meier-Straße 21, 79104 Freiburg, Germany*
[7]*Fraunhofer Institute for Solar Energy Systems, Heidenhofstr. 2, 79110 Freiburg, Germany*
[8]*Bavarian Research Institute of Experimental Geochemistry and Geophysics, University of Bayreuth, Universitätsstr. 30, D-95440 Bayreuth, Germany*
[9]*Deutsches Elektronen-Synchrotron (DESY), Notkestr. 85, 22607 Hamburg, Germany*
[10]*Forschungsneutronenquelle Heinz Maier-Leibnitz FRM-II, Technische Universität München, Lichtenbergstr. 1, D-85747 Garching near München, Germany*
[11]*Institut für Werkstoffwissenschaft, Technische Universität Dresden, Helmholtzstr. 7, D-01062 Dresden, Germany*
[12]*Karlsruhe Institute of Technology (KIT), Institute for Applied Materials (IAM), Hermann-von-Helmholtz-Platz 1, D-76344 Eggenstein-Leopoldshafen, Germany*
[13]*Felix Bloch Institute for Solid-State Physics, Leipzig University, 04103 Leipzig, Germany*
[14]*Experimental Physics VI, Center for Electronic Correlations and Magnetism, University of Augsburg, 86135 Augsburg, Germany*

(*) Corresponding e-mail: d.mikhailova@ifw-dresden.de

(#) date of death 23.04.2024

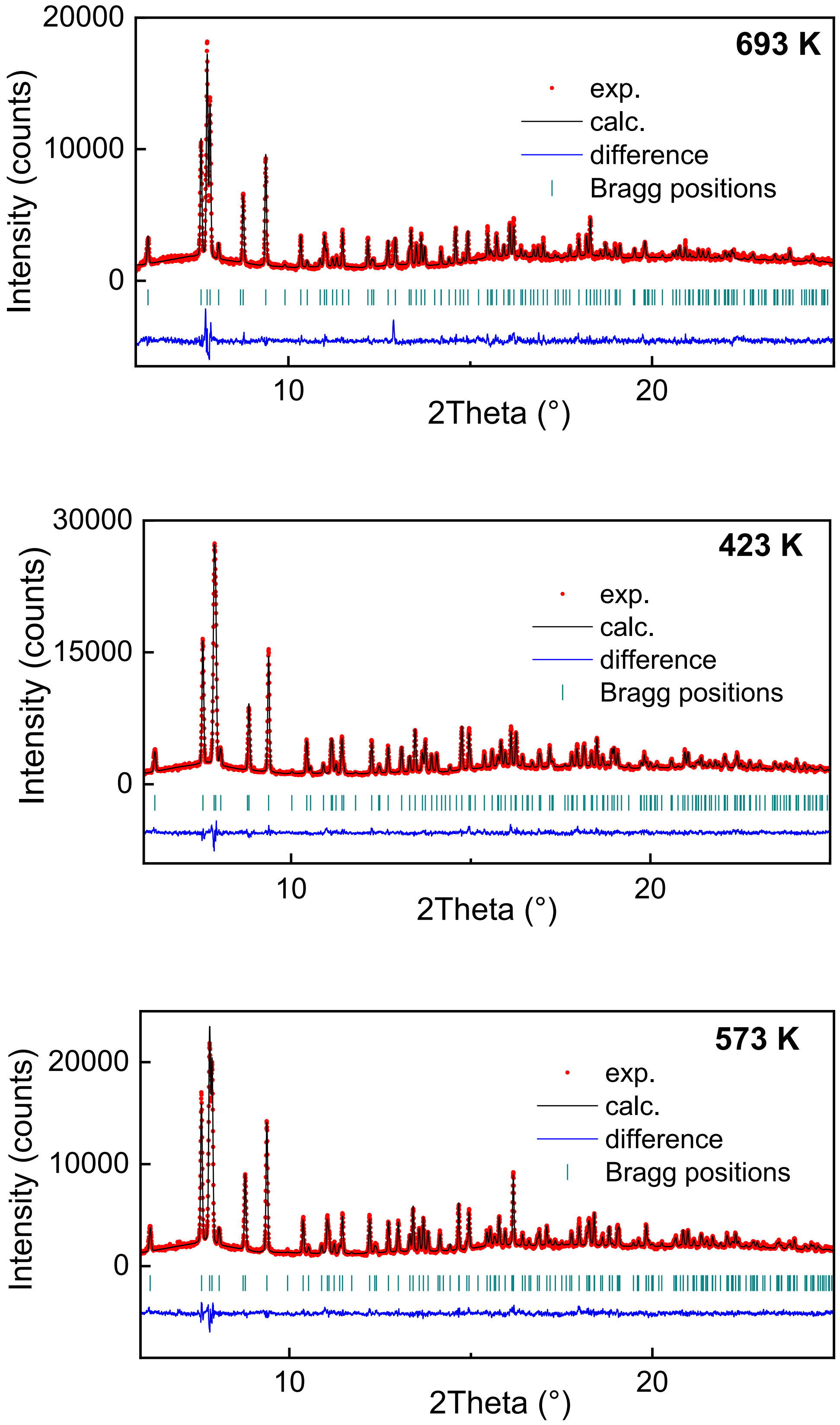

Figure S1. Examples of synchrotron powder diffraction patterns for the ambient-pressure $CuReO_4$ modification together with Rietveld analysis of the data, at three different temperatures. For Rietveld analysis, the room-temperature structural model[20] was applied.

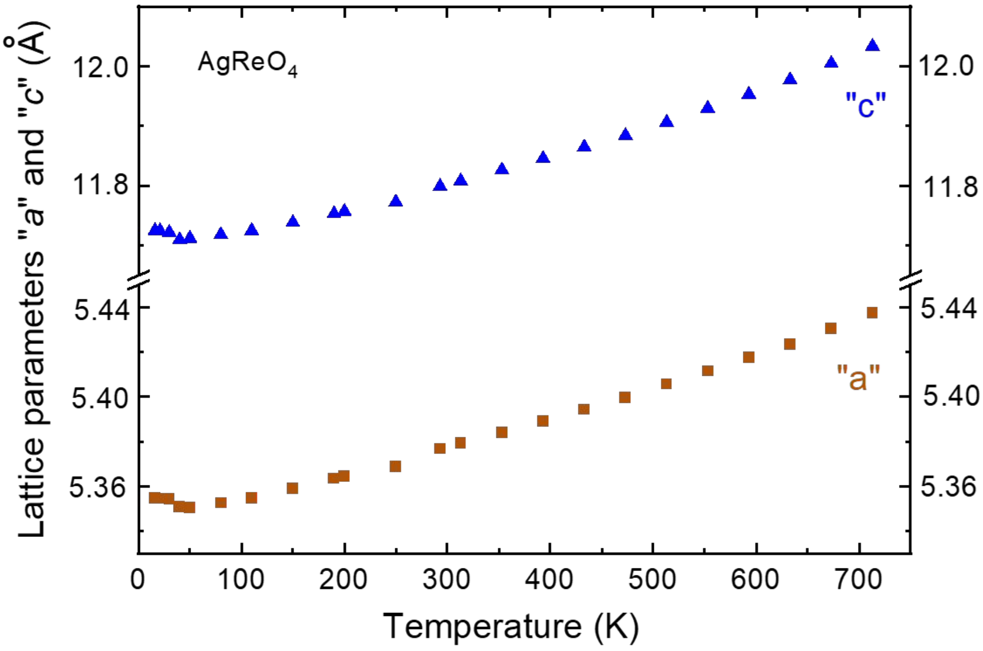

Figure S2. Temperature dependence of the AgReO4 lattice parameters at ambient pressure, obtained from the synchrotron powder diffraction measurements, together with a linear fitting in the range of 100-710 K.

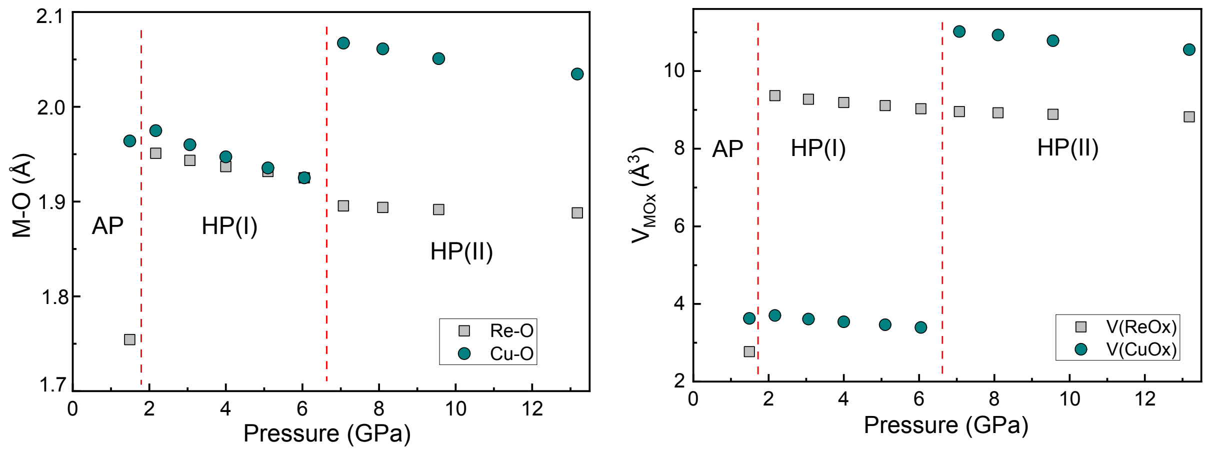

Figure S3. Pressure dependence of metal-oxygen distances and polyhedron volumes in $CuReO_4$ polymorphs, from the DFT calculations. Whereas in $CuReO_4$-AP, Re and Cu have a distorted tetrahedral oxygen surrounding, rhenium cations adopt octahedra in $CuReO_4$-HP(I), and both cations Re and Cu exhibit octahedral environment in $CuReO_4$-HP(II) with a NbO_2-type structure.

Table S1. DFT/PAW Γ-point phonon frequencies for the ambient-pressure $CuReO_4$ phase (space group $I4_1cd$, point group $C4v$).

	IDs	Energy, cm^{-1}	Sym(A)			IDs	Energy, cm^{-1}	Sym(A)
**4**	6	8.0	A1(R)		**56**	75 76	268.0	E(R)
**5**	7 8	22.0	E(R)		**57**	77 78	273.0	E(R)
**6**	9	26.0	B1(R)		**58**	79	285.0	B2(R)
**7**	10 11	35.0	E(R)		**59**	80 81	286.0	E(R)
**8**	12	41.0	B1(R)		**60**	82	289.0	A1(R)
**9**	13	42.0	A2(IR)		**61**	83	290.0	B1(R)
**10**	14	43.0	B2(R)		**62**	84	291.0	A2(IR)
**11**	15 16	44.0	E(R)		**63**	85 86	292.0	E(R)
**12**	17 18	51.0	E(R)		**64**	87	295.0	B1(R)
**13**	19	51.0	A1(R)		**65**	88	298.0	B2(R)
**14**	20	52.0	B2(R)		**66**	89 90	299.0	E(R)
**15**	21 22	55.0	E(R)		**67**	91	306.0	B1(R)
**16**	23	56.0	B1(R)		**68**	92	307.0	A1(R)
**17**	24	65.0	A2(IR)		**69**	93 94	308.0	E(R)
**18**	25	69.0	B2(R)		**70**	95	311.0	B2(R)
**19**	26	73.0	A2(IR)		**71**	96	311.0	A2(IR)
**20**	27	76.0	B1(R)		**72**	97 98	313.0	E(R)
**21**	28	80.0	A2(IR)		**73**	99	324.0	A1(R)
**22**	29	82.0	A1(R)		**74**	100 101	325.0	E(R)
**23**	30 31	83.0	E(R)		**75**	102	331.0	B1(R)
**24**	32	84.0	B2(R)		**76**	103	333.0	A2(IR)
**25**	33	88.0	B1(R)		**77**	104	333.0	B1(R)
**26**	34 35	91.0	E(R)		**78**	105	335.0	B2(R)
**27**	36 37	93.0	E(R)		**79**	106	339.0	B2(R)
**28**	38 39	100.0	E(R)		**80**	107	340.0	A2(IR)
**29**	40	100.0	A2(IR)		**81**	108	344.0	A1(R)
**30**	41	107.0	A1(R)		**82**	109 110	346.0	E(R)
**31**	42	113.0	A1(R)		**83**	111 112	349.0	E(R)
**32**	43 44	119.0	E(R)		**84**	113	856.0	B1(R)
**33**	45	120.0	B2(R)		**85**	114 115	859.0	E(R)
**34**	46	127.0	B1(R)		**86**	116	859.0	A1(R)
**35**	47	129.0	B2(R)		**87**	117	862.0	A2(IR)
**36**	48 49	133.0	E(R)		**88**	118 119	866.0	E(R)
**37**	50	136.0	A2(IR)		**89**	120	866.0	A1(R)
**38**	51 52	138.0	E(R)		**90**	121	868.0	B1(R)
**39**	53	147.0	A1(R)		**91**	122	868.0	B2(R)
**40**	54	154.0	A1(R)		**92**	123	873.0	A2(IR)
**41**	55 56	157.0	E(R)		**93**	124	875.0	B2(R)
**42**	57	163.0	A2(IR)		**94**	125 126	878.0	E(R)
**43**	58 59	166.0	E(R)		**95**	127 128	879.0	E(R)
**44**	60	173.0	B1(R)		**96**	129	883.0	B2(R)
**45**	61	179.0	B2(R)		**97**	130	889.0	A1(R)
**46**	62 63	183.0	E(R)		**98**	131 132	891.0	E(R)
**47**	64	184.0	B1(R)		**99**	133 134	893.0	E(R)
**48**	65	206.0	B2(R)		**100**	135	894.0	B1(R)
**49**	66	207.0	B1(R)		**101**	136	897.0	A2(IR)
**50**	67 68	219.0	E(R)		**102**	137	920.0	B2(R)
**51**	69	233.0	A2(IR)		**103**	138	925.0	B1(R)
**52**	70 71	234.0	E(R)		**104**	139 140	927.0	E(R)
**53**	72	244.0	A1(R)		**105**	141 142	930.0	E(R)
**54**	73	257.0	A1(R)		**106**	143	935.0	A1(R)
**55**	74	263.0	A2(IR)		**107**	144	937.0	A2(IR)

Analysis of Raman $CuReO_4$-HP(I) spectra

The high-pressure (HP) $CuReO_4$ phases (space group $I4_1/a$) possess the point symmetry group $C_{4h}(4/m)$. The 144 phonon modes at the Γ-point have the following irreducible representations:

$\Gamma(C_{4h}) = 18A_g(R) + 18A_u(IR) + 18B_g(R) + 18B_u(IR) + 36E_g(R) + 36E_u(IR)$,

where E_u, A_u, B_u modes are IR-active.

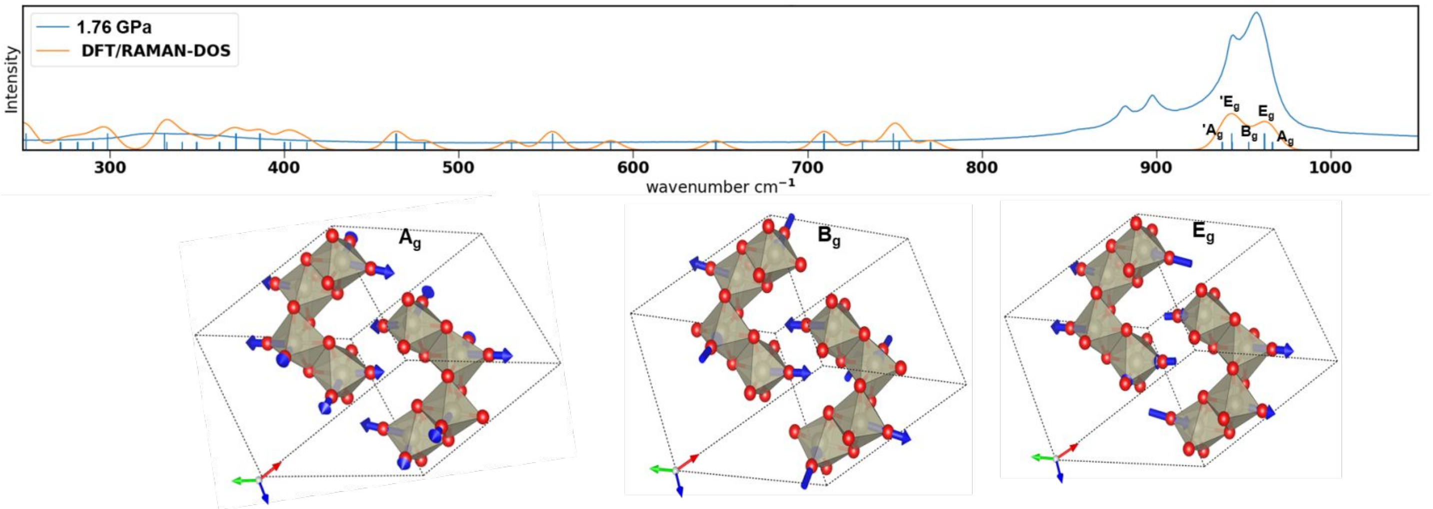

Figure S4. Raman spectrum of $CuReO_4$-HP(I) at 1.76 GPa compared to DFT phonon density of states. The lower panel shows displacement vector orientation for representative modes, such as symmetric and asymmetric stretching vibration of Re–O bonds (A_g, E_g, B_g) in ReO_6 polyhedra.

Table S2 summarizes the high-frequency part of the spectrum for $CuReO_4$-HP(I) together with the experimentally observed features. In $CuReO_4$-HP(I), similar to $CuReO_4$-AP, the higher frequency components are mostly dominated by stretching vibrations occurring around 950 cm^{-1}. Co-related symmetry elements of the AP phase (A_1, B_2, and E) transform to E_g and A_g modes of the high-pressure phase. A multi-mode band at 900 cm^{-1} for the AP phase totally disappears at the equilibrium geometry of the HP(I) phase.

Table S2. DFT-computed and experimental vibrational-frequencies of $CuReO_4$-HP(I) in the Γ-point and the description of vibrational modes.

$CuReO_4$-HP(I), $I4_1/a$			
Sym.	Calc. DFT	Raman[a]	Mode[b]
A_g(133)	912	943(s,br)	ν(Re–O), asym
E_g(134,135)	917		ν(Re–O)
B_g(136)	918		ν(Re–O)
B_g(139)	927	950(sh)	ν(Re–O), asym
E_g(141,142)	936		ν(Re–O), asym

$A_g(144)$	940	957(vs)	ν(Re–O), sym

[a]Vibrational frequencies are in cm^{-1}, relative intensities are: 'vs' - very strong, 's'- strong, 'sh' - shoulder, 'br' - broad.
[b]The mode description highlights the main type of contributing coordinates, but the overall mode composition is usually more complex. Abbreviations: '*ν*(Re–O)' - symmetric and asymmetric stretching vibration of Re–O bonds.

The most pronounced difference between theoretical predictions in $CuReO_4$-HP(I) when comparing to the high-pressure experiment at 1.76 GPa is that three distinct peaks between 850-900 are missing. The plausible reason lies in the fact that the theoretical models consider for vibration analysis using the equilibrium geometry of $CuReO_4$-HP(I) as predicted at DFT level, while all experimentally observed spectra is $CuReO_4$-HP(I) phase under some pressure.
